# Association of the apolipoprotein A5 gene -1131 T>C polymorphism with fasting blood lipids: a meta-analysis in 37859 subjects

**DOI:** 10.1186/1471-2350-11-120

**Published:** 2010-08-10

**Authors:** Tongfeng Zhao, Jiangpei Zhao

**Affiliations:** 1 Department of Geriatrics, the Second Affiliated Hospital, School of Medicine, Zhejiang University, 88 Jiefang Road, Hangzhou 310009, PR China; 2 Department of Geriatrics, Hangzhou Hospital of Traditional Chinese Medicine, 453 Tiyuchang Road, Hangzhou 310009, PR China

## Abstract

**Background:**

Studies examining the association of apolipoprotein A5 (APOA5) gene -1131 T>C polymorphism with blood lipids produced inconsistent results. In this meta-analysis encompassing all the relevant studies, we aimed to investigate the association of the -1131 T>C polymorphism with fasting blood lipids.

**Methods:**

We limited our analysis to the following four blood lipid variables: total cholesterol (TC), triglycerides (TG), low density lipoprotein cholesterol (LDL-C), and high-density lipoprotein cholesterol (HDL-C). Subjects were confined to adults who were at least 18 years old. A dominant model was used for this meta-analysis. 37 studies with 37859 subjects were included in this meta-analysis.

**Results:**

The results showed that the carriers of -1131C allele have higher blood TC and TG than the non-carriers: standardized mean difference (SMD) = 0.08, 95% confidence interval (CI, 0.05, 0.11), *P *< 0.00001, *P*_heterogeneity _= 0.42, and SMD = 0.31, 95% CI (0.27, 0.34), *P *< 0.00001, *P*_heterogeneity _= 0.0003, respectively. Significant association between the -1131 T>C polymorphism and lower blood HDL-C was also detected under the dominant model: SMD = -0.17, 95% CI (-0.21, -0.14), *P *< 0.00001, *P*_heterogeneity _= 0.003.

**Conclusions:**

Our meta-analysis supports the strong association of the APOA5 -1131 T>C polymorphism with higher levels of TC and TG, and lower levels of HDL-C.

## Background

Hyperlipidemia, which is considered to be one of the most important risk factors for coronary heart disease (CHD) and stroke, is characterized by the derangements of one or many of the lipids: elevations of total cholesterol (TC), low density lipoprotein cholesterol (LDL-C) and/or triglycerides (TG), or low levels of high-density lipoprotein cholesterol (HDL-C) [1]. Although a large number of studies have tried to elucidate the pathogenesis of the disease, the exact underlying mechanisms are still not completely understood [2]. In recent years, much has been learned about specific genes that influence hyperlipidemia [3]. However, due to various reasons, including considerable heterogeneity of the disease, the identification of susceptibility genes is difficult and most associations have not been replicated [3].

More recently, apolipoprotein A5 (APOA5) was identified as a strong modulator of blood lipids [4]. The APOA5 is predominantly synthesized in the liver and secreted into the plasma where it plays a central role in regulating TG metabolism [4]. A higher plasma APOA5 would result in lower TG levels [5]. APOA5 knockout mice develop hypertriglyceridemia, whereas transgenic mice overexpressing APOA5 have low TG levels [5]. APOA5 reduces plasma TG by inhibiting very low density lipoprotein-TG production and stimulating lipoprotein lipase-mediated very low density lipoprotein-TG hydrolysis [6]. APOA5 also play important roles in modulating other blood lipid metabolism [6-8]. Several studies have demonstrated that the APOA5 gene polymorphisms are associated with reduced HDL-C levels and decreased low density lipoprotein particle size [6-10]. Given its role in blood lipid metabolism, the APOA5 gene is considered a candidate gene for hyperlipidemia.

The APOA5 gene is located on chromosome 11q23 within the APOA1/C3/A4/A5 gene cluster, and comprises 4 exons encoding 366 amino acids [5,11]. A number of human APOA5 gene nucleotide polymorphisms (SNPs) have been investigated for a possible role in mediating genetic predisposition to hyperlipidemia [11]. The most extensively studied polymorphism is APOA5 -1131 T>C polymorphism (rs662799, SNP3). This polymorphism is located in the promoter region of the APOA5 gene [12]. A number of investigators studied the possible association between this polymorphism and blood lipids, but the results are conflicting and inconclusive [6-42]. In this paper, a meta-analysis was performed on previous reports to investigate the association of the APOA5 -1131 T>C polymorphism with fasting blood lipids.

## Methods

### Identification and eligibility of relevant studies

We identified all articles published before November 2009 on the APOA5 -1131 T>C polymorphism and its association with blood lipids. A systematic search of the literature was carried out by using PubMed and HugeNavigator. The language was limited to English. The keywords used for this search were "APOAV OR APOA-V OR apolipoprotein A-V OR apolipoprotein AV OR apolipoprotein A5 OR APOA5 OR APO A5" concatenated with "polymorphism OR variant OR SNP OR mutation". We limited our analysis to the following four blood lipid variables: TC, TG, LDL-C, and HDL-C. The selection criteria for studies to be considered for this meta-analysis were as follows: (1) data were reported on at least 1 of the four blood lipid variables; (2) data reported on fasting blood lipid; (3) in case of interventional studies, we used pre-intervention baseline data; (4) we only included studies in which mean blood lipid levels and standard deviations (SD) or standard errors by genotype were available; (5) subjects were confined to adults who were at least 18 years old. All references cited in the studies were also reviewed in order to find other published work that was not indexed by PubMed and HugeNavigator. Animal studies, case reports, review articles, abstracts, reports with incomplete data, and studies based on pedigree data were excluded.

### Data extraction

Two investigators independently reviewed the articles to exclude irrelevant and overlapping studies. The results were compared, and disagreements were discussed and resolved by consensus. When overlapping articles were found, we only included the publication that reported the most extensive information. From each study, the following information was extracted: journal, year of publication, first author, demographics, racial background of the study population, fasting status, age, sex, health condition, sample size, mean and SD or standard error by genotypes, genotyping and lipid assay methods, unit of the four lipid variables.

### Statistical analysis

All data in this analysis were presented as mean ± SD. When the standard error was reported in the original article, the value of the SD was calculated. Review Manager 5.0 software (The Cochrane Collaboration, Oxford, UK) was used for the meta-analysis. Because the frequencies of the minor allele homozygous were low, to ensure adequate statistical power, we employed a dominant model [(TC+CC) versus TT] for this meta-analysis. When information was reported for more than one subpopulation (for example, male subjects or female subjects, subjects with type 2 diabetes and control subjects, subjects from different geographical areas or different ethnicity) in one study, each subpopulation was treated as a separate comparison in our meta-analysis. In addition, we conducted subgroup analyses by ethnicity, gender, and health condition. Ethnic subgroups were defined as European descendents, East Asian, or populations of other ethnic origins. Considering that the effects of APOA 5 gene on blood lipids may be influenced by lipid-lowering therapy, we also performed a subgroup analysis in subjects without taking lipid-lowering medication. The healthy subjects (normal subjects or healthy subjects) and the subjects who ceased lipid-lowering therapy at least 1 month were also included in this subgroup. For TG, we also conducted a larger study (with sample size more than five hundreds) subgroup analysis. To ensure adequate statistical power, we only performed the meta-analysis on the subgroup with at least four studies.

The existence of heterogeneity between studies was ascertained by Q-statistic. Heterogeneity was considered significant for *P *< 0.1. A pooled standardized mean difference (SMD), together with 95% confidence interval (CI), was used for this meta-analysis. The SMD was chosen because the blood lipids were measured using different scanners [43]. Because a random effects model considers both between-study and within-study heterogeneity, it provides a more conservative evaluation of the significance of the association than one based on fixed effects [44]. The random effects model was used for this meta-analysis.

All populations described in the studies were tested for Hardy-Weinberg equilibrium (HWE). We repeatedly performed the meta-analysis via excluding the studies not in HWE to test the stability of the results.

Galbraith plot, made by MIX 1.7 software (Kitasato Clinical Research Center, Kitasato University, Japan), was used to detect potential sources of heterogeneity [45,46]. The pooled SMDs were recalculated after removal of the outlier studies identified in the plot.

Funnel plot was performed to look for evidence of publication bias. The funnel plot should be asymmetric when there is publication bias and symmetric in case of no publication bias. Egger's test, estimated by MIX 1.7 software was performed to measure the funnel plot asymmetry [45-47]. A significance level of 0.1 was used as an indication for the presence of potential publication bias. A *P *< 0.1 indicates the presence of publication bias.

## Results

### Selection and characteristics of studies

Additional file 1 (figure legend of additional file 1 is in additional file 2) describes the flow of candidate and eligible papers. Initial search of the literature yielded 220 publications. We excluded 91 irrelevant papers on the basis of title and abstract. The original papers were retrieved and evaluated for compliance with the inclusion criteria. 92 papers were ineligible for the following reasons: 43 papers did not provide complete data for this meta-analysis, 29 papers presented data on other polymorphisms, 8 papers had subjects overlap with other publications, 6 studies were based on pedigree data, 5 papers were review articles, and 1 paper involved subjects younger than 18 years.

The selected study characteristics were summarized in Table S1 of additional file 3. A total of 37 studies were included in this meta-analysis. Of these, 34, 27, 23, and 30 studies separately presented the data on TG, TC, LDL-C, and HDL-C. In the 37 studies, there were 13 studies of European descendents, 15 studies of East Asians, 5 studies of other ethnic origins. 4 studies reported results on different racial descendent population and each population was treated as a separate comparison in the meta-analysis. In 1 study, all the subjects were female, and in 5 studies, all the subjects were male; while in the remaining 31 studies, the subjects consisted of both male and female; among the 31 studies, 4 studies provided the data on male and female subjects respectively. In the eligible studies, 6 studies involved subjects with type 2 diabetes, 9 studies involved healthy subjects, and 6 studies involved subjects with CHD. 19 studies clearly stated that the overall populations or one/several subpopulations did not take lipid-lowering medication. In all 37 studies, there were 19 studies separately providing the information on more than one subpopulation. Each subpopulation was treated as a separate comparison. Genotype distribution in 3 subpopulations from 3 studies significantly deviated from the expected HWE. The units of blood lipids used in the eligible studies included mg/dl and mmol/L. The complete blood lipids data by genotypes can be found in Table S2 of additional file 3.

### Summary statistics

We distinguished 66 comparisons on the basis of categories like ethnicity, gender, and health condition. Of these, 60, 42, 37, and 48 comparisons were included for comparing the difference in blood TG, TC, LDL-C, and HDL-C, respectively (Table S2 of additional file 3, Table 1). Overall, 37859 subjects were enrolled in this meta-analysis. Among them, 24226 subjects (64%) had the genotype TT, and 13633 (36%) were carriers of -1131C allele (Table S2 of additional file 3). 34193, 20961, 19860, and 29588 subjects were included in comparing the difference in blood TG, TC, LDL-C, and HDL-C, respectively (Table S2 of additional file 3).

**Table 1:** Meta-analysis of the APOA5 -1131T>C polymorphism and blood lipids association

Group and subgroups under analysis	Comparisons (n)	Q test *P *value	SMD (95% CI)	*P*
TC				
All	42	0.42	0.08 (0.05, 0.11)	<0.00001
All in HWE	39	0.30	0.08 (0.04, 0.12)	0.0001
No lipid-lowering medicine	20	0.47	0.08 (0.03, 0.14)	0.003
No lipid-lowering medicine in HWE	19	0.41	0.08 (0.02, 0.14)	0.005
European	16	0.16	0.07 (-0.00, 0.15)	0.05
European in HWE	14	0.09	0.07 (-0.02, 0.16)	0.11
East Asian	18	0.69	0.09 (0.05, 0.13)	<0.0001
East Asian in HWE	17	0.63	0.09 (0.04, 0.15)	0.0003
Other	8	0.45	0.04 (-0.03, 0.11)	0.23
Healthy	8	0.45	0.04 (-0.04, 0.13)	0.31
Type 2 diabetes	7	0.64	0.11 (-0.01, 0.23)	0.07
CHD	6	0.71	0.06 (-0.03, 0.15)	0.18
CHD in HWE	5	0.58	0.06 (-0.04, 0.15)	0.22
Male	6	0.29	0.02 (-0.09, 0.13)	0.67
Male in HWE	5	0.20	0.03 (-0.12, 0.17)	0.74
TG				
All	60	0.0003	0.31 (0.27, 0.34)	<0.00001
All in HWE	58	0.004	0.31 (0.28, 0.35)	<0.00001
No lipid-lowering medicine	28	0.35	0.33 (0.29, 0.38)	<0.00001
No lipid-lowering medicine in HWE	27	0.32	0.33 (0.29, 0.38)	<0.00001
European	21	0.29	0.36 (0.29, 0.43)	<0.00001
European in HWE	20	0.26	0.36 (0.29, 0.43)	<0.00001
East Asian	23	0.001	0.34 (0.29, 0.39)	<0.00001
East Asian in HWE	22	0.10	0.35 (0.30, 0.39)	<0.00001
Other	16	0.27	0.21 (0.16, 0.26)	<0.00001
Healthy	8	0.89	0.36 (0.27, 0.44)	<0.00001
Type 2 diabetes	7	0.77	0.40 (0.29, 0.52)	<0.00001
CHD	6	0.88	0.39 (0.29, 0.48)	<0.00001
CHD in HWE	5	0.81	0.38 (0.28, 0.48)	<0.00001
Male	10	0.39	0.26 (0.19, 0.33)	<0.00001
Female	7	0.13	0.21 (0.11, 0.31)	<0.0001
Larger	25	0.009	0.26 (0.22, 0.30)	<0.00001
Larger in HWE	24	0.05	0.27 (0.23, 0.31)	<0.00001
LDL-C				
All	37	0.010	0.03 (-0.02, 0.07)	0.22
All in HWE	34	0.009	0.02 (-0.02, 0.07)	0.34
No lipid-lowering medicine	16	0.32	0.02 (-0.05, 0.08)	0.61
No lipid-lowering medicine in HWE	15	0.26	0.02 (-0.05, 0.09)	0.58
European	11	0.01	-0.00 (-0.10, 0.10)	0.99
European in HWE	9	0.005	-0.01 (-0.14, 0.12)	0.93
East Asian	20	0.04	0.04 (-0.01, 0.10)	0.14
East Asian in HWE	19	0.05	0.04 (-0.03, 0.10)	0.25
Other	6	0.44	0.02 (-0.06, 0.09)	0.68
Healthy	8	0.03	0.05 (-0.09, 0.18)	0.50
Type 2 diabetes	8	0.11	0.04 (-0.09, 0.16)	0.55
CHD	5	0.73	-0.03 (-0.12, 0.07)	0.55
CHD in HWE	4	0.57	-0.03 (-0.13, 0.07)	0.56
Male	5	0.02	-0.06 (-0.25, 0.13)	0.51
Male in HWE	4	0.02	-0.09 (-0.34, 0.15)	0.47
HDL-C				
All	48	0.003	-0.17 (-0.21, -0.14)	<0.00001
All in HWE	45	0.003	-0.17 (-0.21, -0.14)	<0.00001
No lipid-lowering medicine	24	0.003	-0.20 (-0.27, -0.13)	<0.00001
No lipid-lowering medicine in HWE	23	0.002	-0.20 (-0.28, -0.13)	<0.00001
European	13	0.03	-0.15 (-0.24, -0.06)	0.002
European in HWE	11	0.01	-0.16 (-0.27, -0.05)	0.006
East Asian	25	0.07	-0.20 (-0.24, -0.16)	<0.00001
East Asian in HWE	24	0.07	-0.19 (-0.24, -0.15)	<0.00001
Other	10	0.13	-0.14 (-0.22, -0.06)	0.0008
Healthy	8	0.29	-0.17 (-0.26, -0.07)	0.0007
Type 2 diabetes	9	0.36	-0.18 (-0.28, -0.09)	0.0002
CHD	5	0.003	-0.21 (-0.42, -0.01)	0.06
CHD in HWE	4	0.002	-0.24 (-0.48, -0.01)	0.06
Male	6	0.55	-0.11 (-0.20, -0.02)	0.02
Male in HWE	5	0.41	-0.11 (-0.21, -0.00)	0.05

### Association between the APOA5 -1131 T>C polymorphism and TC

**Figure 1:**
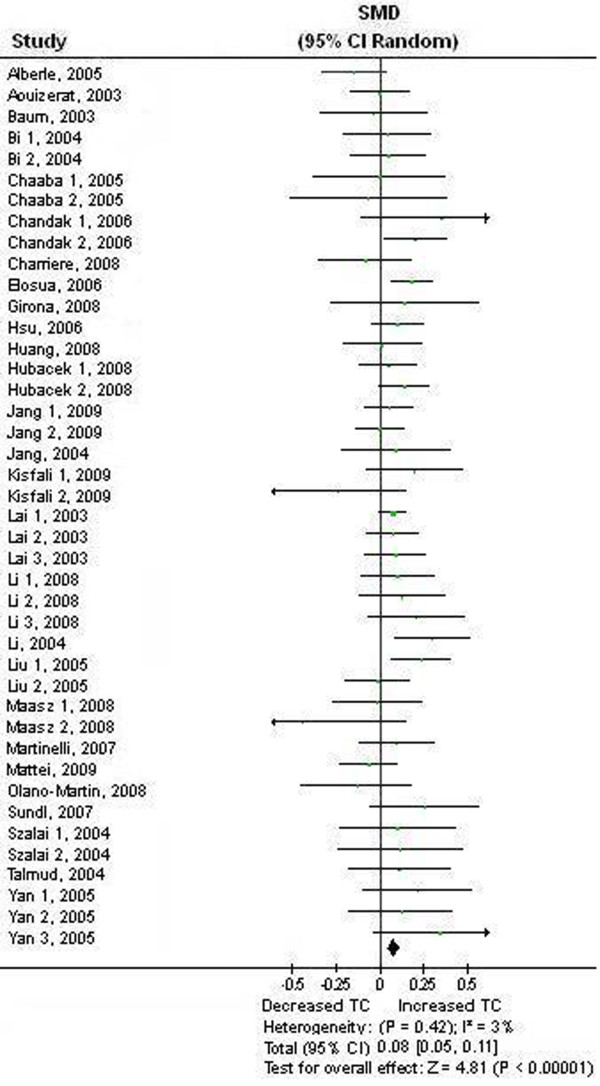
**Forest plot of the APOA5 -1131 T>C polymorphism and TC association**. Result from the analysis on all 42 comparisons.

We first performed the meta-analysis on all 42 comparisons. The result showed that the carriers of -1131C allele have higher blood TC than the non-carriers: SMD = 0.08, 95% CI (0.05, 0.11), *P *< 0.00001, *P*_heterogeneity _= 0.42 (Table 1, Figure 1). We then performed the subgroup analyses stratified by the characteristics of the subjects. The significant association between the APOA5 -1131 T>C polymorphism and blood TC in subjects without taking lipid-lowering medication was also detected under the dominant models: SMD = 0.08, 95% CI (0.03, 0.14), *P *= 0.003, *P*_heterogeneity _= 0.47 (Table 1). The association between the APOA5 -1131 T>C polymorphism and higher levels of TC under dominant model was significant in East Asians, and marginally significant in European descendents: SMD = 0.09, 95% CI (0.05, 0.13), *P *< 0.0001, *P*_heterogeneity _= 0.69, and SMD = 0.07, 95% CI (-0.00, 0.15), *P *= 0.05, respectively (Table 1). The other pooled SMDs from the subgroup analyses were not significant (*P*>0.05) (Table 1).

We next conducted the analyses excluding the studies not in HWE (Table 1). The association between the APOA5 -1131 T>C polymorphism and higher levels of TC under dominant model was not significant in European descendents: SMD = 0.07, 95% CI (-0.02, 0.16), *P *= 0.11, *P*_heterogeneity _= 0.09. The other results were similar to those when the studies not in HWE were included.

### Association between the APOA5 -1131 T>C polymorphism and TG

**Figure 2:**
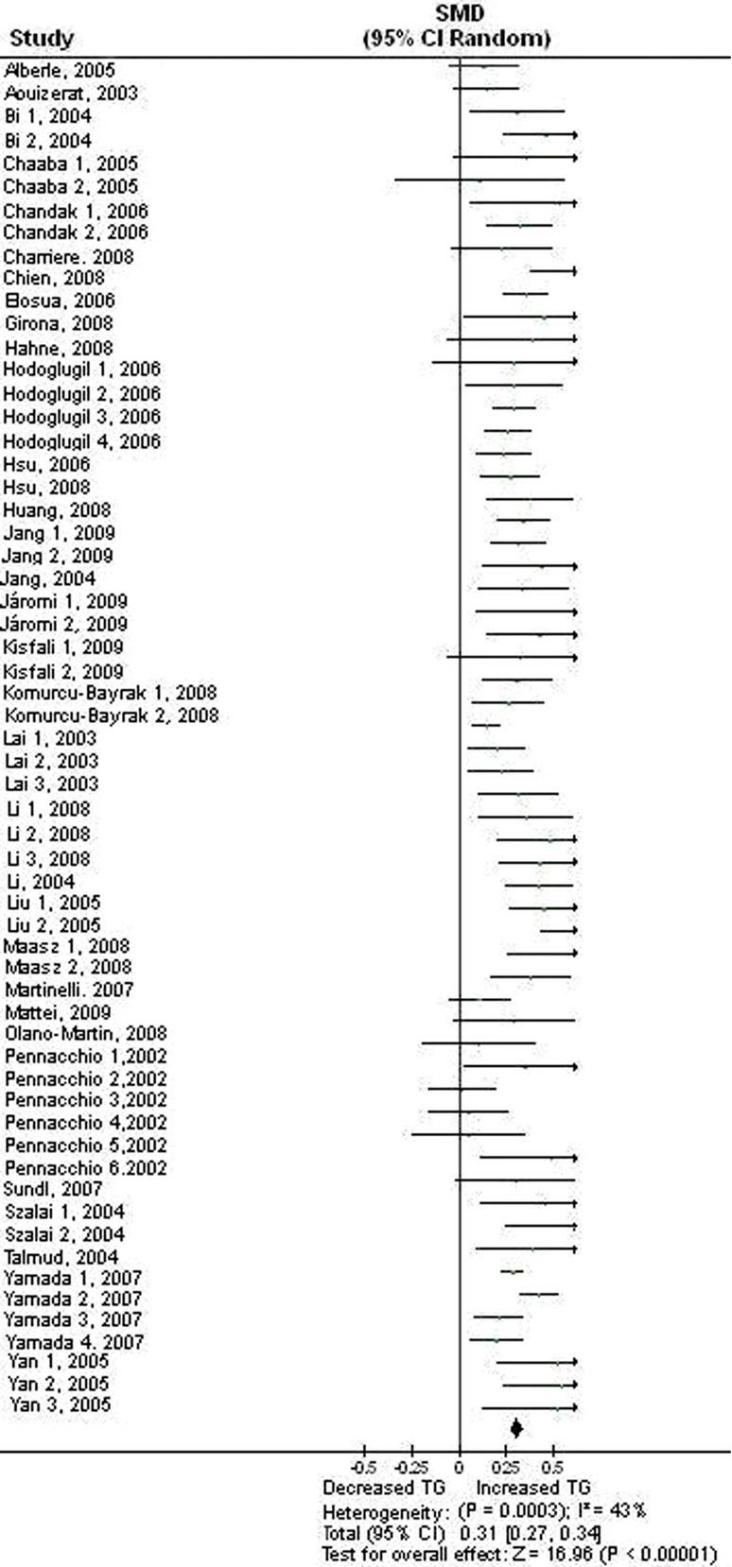
**Forest plot of the APOA5 -1131 T>C polymorphism and TG association**. Result from the analysis on all 60 comparisons.

The result from the meta-analysis on all 60 comparisons showed that the carriers of -1131C allele have higher blood TG than the non-carriers in overall population: SMD = 0.31, 95% CI (0.27, 0.34), *P *< 0.00001, *P*_heterogeneity _= 0.0003 (Table 1, Figure 2). The association between the APOA5 -1131 T>C polymorphism and higher levels of TG under dominant model was also significant across all subpopulations (*P *< 0.0001) (Table 1).

There was significant heterogeneity among the available studies. However, the heterogeneity was effectively decreased or removed in the subgroups stratified by the characteristics of the subjects (Table 1).

### Association between the APOA5 -1131 T>C polymorphism and LDL-C

**Figure 3:**
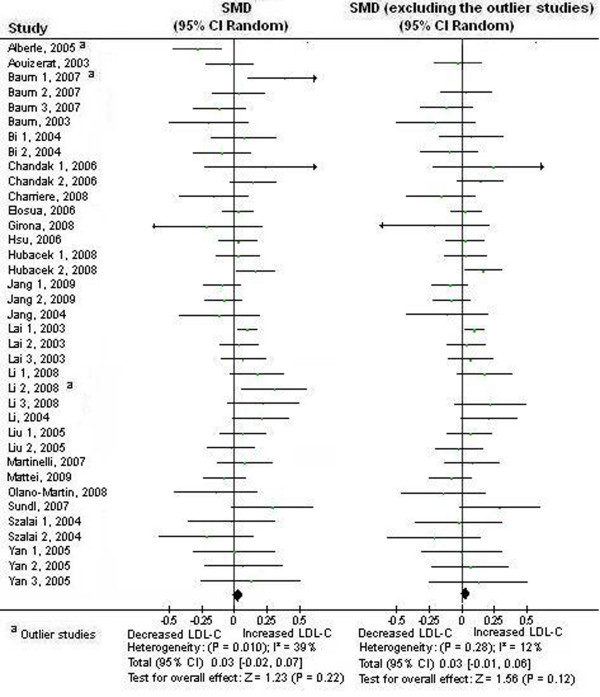
**Forest plot of the APOA5 -1131 T>C polymorphism and LDL-C association**. Result from the analysis on all 37 comparisons. There was significant heterogeneity among the studies. The pooled SMD was recalculated after removal of the outlier studies.

The results from our meta-analysis did not suggest a significant association between the -1131 C>T polymorphism and higher levels of LDL-C under dominant model (*P*>0.05) (Table 1, Table 2, Figure 3).

**Table 2:** Meta-analysis of the APOA5 -1131T>C polymorphism and blood lipids association (excluding the outlier studies)

Group and subgroups under analysis	Comparisons (n)	Q test *P *value	SMD (95% CI)	*P*
LDL-C				
All	34	0.28	0.03 (-0.01, 0.06)	0.12
All in HWE	31	0.28	0.02 (-0.02, 0.06)	0.34
No lipid-lowering medicine	15	0.76	-0.00 (-0.06, 0.06)	0.94
No lipid-lowering medicine in HWE	14	0.69	-0.00 (-0.06, 0.06)	0.96
European	10	0.26	0.05 (-0.04, 0.13)	0.27
European in HWE	8	0.14	0.05 (-0.06, 0.16)	0.38
East Asian	18	0.24	0.02 (-0.03, 0.07)	0.37
East Asian in HWE	17	0.40	0.00 (-0.05, 0.06)	0.87
Healthy	7	0.18	-0.00 (-0.11, 0.11)	0.98
Type 2 diabetes	7	0.36	-0.01 (-0.11, 0.10)	0.89
Male	4	0.14	0.01 (-0.17, 0.19)	0.94
Male in HWE	3	0.07	-0.01 (-0.30, 0.29)	0.96
HDL-C				
All	44	0.55	-0.15 (-0.18, -0.13)	<0.00001
All in HWE	41	0.66	-0.15 (-0.17, -0.12)	<0.00001
No lipid-lowering medicine	21	0.66	-0.15 (-0.20, -0.10	<0.00001
No lipid-lowering medicine in HWE	20	0.61	-0.15 (-0.20, -0.10)	<0.00001
European	12	0.46	-0.11 (-0.17, -0.05)	0.0005
European in HWE	10	0.29	-0.12 (-0.20, -0.04)	0.005
East Asian	23	0.71	-0.18 (-0.21, -0.15)	<0.00001
East Asian in HWE	22	0.80	-0.17 (-0.20, -0.13)	<0.00001
Other	9	0.74	-0.10 (-0.16, -0.03)	0.004
CHD	4	0.61	-0.12 (-0.23, -0.01)	0.04
CHD in HWE	3	0.42	-0.12 (-0.24, -0.01)	0.04

There was significant heterogeneity among the available studies. Significant heterogeneity was also observed in European subgroup, East Asian subgroup, healthy subject subgroup, and male subject subgroup (Table 1). 3 studies (Alberle, 2005, Baum 1, 2007, and Li 2, 2008) were identified as the main contributors of heterogeneity by using the Galbraith plot [Table S1 of additional file 3, additional file 4A (Figure legend is in additional file 2)] [20,35]. The heterogeneity was effectively removed or decreased after exclusion of these outlier studies (Table 2).

### Association between the APOA5 -1131 T>C polymorphism and HDL-C

**Figure 4:**
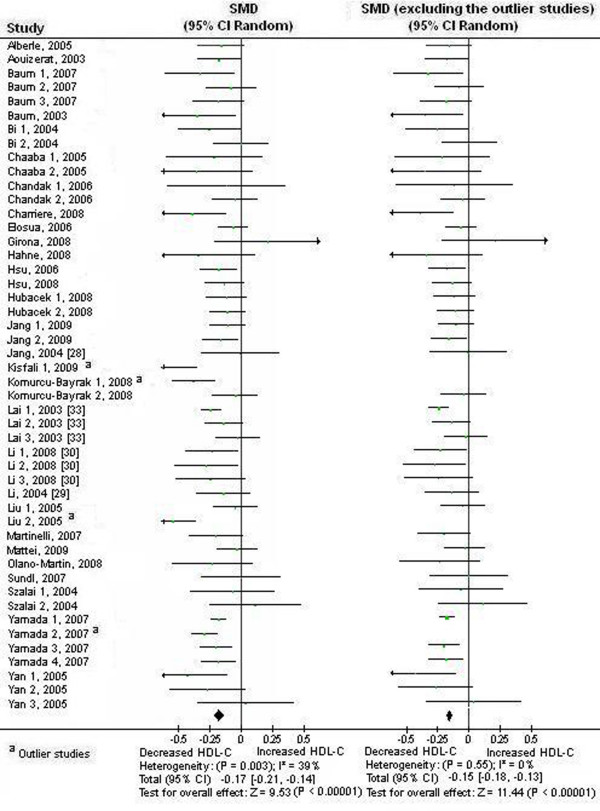
**Forest plot of the APOA5 -1131 T>C polymorphism and HDL-C association**. Result from the analysis on all 48 comparisons. There was significant heterogeneity among the studies. The pooled SMD was recalculated after removal of the outlier studies.

We first performed the meta-analysis on all 48 comparisons. The result showed that the carriers of -1131C allele have lower blood HDL-C than the non-carriers: SMD = -0.17, 95% CI (-0.21, -0.14), *P *< 0.00001, *P*_heterogeneity _= 0.003 (Table 1, Figure 4). We then performed the subgroup analyses stratified by the characteristics of the subjects. All findings from these analyses were significant (*P *< 0.05) except that the association between the APOA5 -1131 T>C polymorphism and lower blood HDL-C under dominant model was marginally significant in CHD subjects: SMD = -0.21, 95% CI (-0.42, -0.01), *P *= 0.06, *P*_heterogeneity _= 0.003 (Table 1).

We next conducted the analyses excluding the studies not in HWE (Table 1). The association between the APOA5 -1131 T>C polymorphism and lower levels of HDL-C under dominant model was marginally significant in male subjects: SMD = -0.11, 95% CI (-0.21, -0.00), *P *= 0.05, *P*_heterogeneity _= 0.41. The remaining results from these analyses were similar to those when the studies not in HWE were included.

There was significant heterogeneity among the available studies. Significant heterogeneity was also observed in European subgroup, East Asian subgroup, subgroup of subjects with CHD, and subgroup of subjects without taking lipid-lowering medicine (Table 1). 4 studies (Kisfali 1, 2009, Komurcu-Bayrak 1, 2008, Liu 2, 2005, and Yamada 2, 2007) were identified as the main contributors of heterogeneity by using the Galbraith plot [Table S1 of additional file 3, additional file 4B (Figure legend is in additional file 2)] [20,35]. The heterogeneity was effectively removed or decreased after exclusion of these outlier studies (Table 2). The association between the APOA5 -1131 T>C polymorphism and lower blood HDL-C under dominant model was significant in CHD subjects: for the analysis including all studies on subjects with CHD: SMD = -0.12, 95% CI (-0.23, -0.01), *P *= 0.04, *P*_heterogeneity _= 0.61, and for the analysis excluding the studies not in HWE: SMD = -0.12, 95% CI (-0.24, -0.01), *P *= 0.04, *P*_heterogeneity _= 0.42 (Table 2). The other results from these analyses were similar to those when the outlier studies were included (Table 2, Figure 4).

### Publication bias

Publication bias was assayed by visual funnel plot inspection and Egger's test. The funnel plots comparing the differences in TC, LDL-C, and HDL-C were all basically symmetric [Additional file 5A, 5B, 5C (Figure legend is in additional file 2)] and Egger's test did not indicate asymmetry of the plots: Intercept = -0.1374, 95% CI (-0.9238, 0.6491), *P *= 0.7259, Intercept = -0.2542, 95% CI (-1.397, 0.8886), *P *= 0.6544, and Intercept = 0.0825, 95% CI (-0.7668, 0.9319), *P *= 0.8458, respectively. The funnel plot comparing the difference in TG showed asymmetry that suggested the existence of publication bias [Additional file 5D (Figure legend is in additional file 2)], and Egger's test did not indicate symmetry of the plots: Intercept = 1.0584, 95% CI (0.3716, 1.7451), *P *= 0.0031. To further evaluate the relationship between the APOA5 -1131 T>C polymorphism and blood TG, we carried the subgroup analysis only including the studies with sample size more than five hundreds. The funnel plot for these studies was basically symmetric [Additional file 5E (Figure legend is in additional file 2)] and Egger's test did not indicate asymmetry of the plot: Intercept = -0.0596, 95% CI (-1.7364, 1.6173), *P *= 0.9421.

## Discussion

The APOA5 -1131 T>C polymorphism has been suggested as a possible genetic factor associated with hyperlipidemia [4,5]. However, the results from the published studies on the association of this polymorphism with hyperlipidemia are conflicting and inconclusive [6-42]. The lack of concordance across many of these studies reflects limitation in the studies, such as small sample sizes, ethnic difference and research methodology.

In this meta-analysis, we investigated the association of the APOA5 -1131 T>C polymorphism with fasting blood lipids. Because the frequencies of the minor allele homozygous were low, most of the studies included in this meta-analysis only provided mean blood lipid levels of all allele C carriers and did not respectively provide mean blood lipid levels of homozygote CC and heterozygote CT. To ensure adequate statistical power, we employed a dominant model for this meta-analysis. The results suggest that the APOA5 -1131 T>C polymorphism under dominant model is significantly associated with fasting blood lipids. The carriers of -1131C allele have higher levels of TC and TG, and lower levels of HDL-C than the non-carriers. In the eligible studies, some studies clearly stated that the overall populations or one/several subpopulations did not take lipid-lowering medication. Because the effects of APOA5 gene on blood lipids may be influenced by lipid-lowering therapy, we performed the subgroup analyses in subjects without taking lipid-lowering medication. The similar association between the -1131 T>C polymorphism and the three blood lipid variables was also detected in these subjects. Since gender, ethnicity, and health condition probably were important variables in determining associative risk with hyperlipidemia, we performed subgroup analyses of gender, ethnicity, and health condition. The effect of this polymorphism on TC especially exists in East Asians, and the effect on TG exists in all subpopulations included in this meta-analysis. For the association of the -1131 T>C polymorphism with lower levels of HDL-C, all findings from this meta-analysis were significant except that the association was marginally significant in male subjects. More studies should be conducted to further examine the association of this polymorphism with HDL-C in male subjects. The association of the -1131 T>C polymorphism with blood HDL-C, TG, and TC was very robust, which did not vary materially when the analyses that removed the studies not in HWE were performed. The significant association of the APOA5 -1131 T>C polymorphism with blood HDL-C was also supported by the results from the meta-analysis performed after removal of the outlier studies. However, our meta-analysis does not suggest the significant association of the APOA5 -1131 T>C polymorphism with blood LDL-C under dominant model. Genome-wide association studies have suggested that the polymorphisms in or near the APOA5 gene are among the strongest known genetic determinants of triglyceride concentration [48,49]. Our findings also are consistent the results from a meta-analysis by Triglyceride Coronary Disease Genetics Consortium and Emerging Risk Factors Collaboration, in which they reported that the APOA5 -1131 T>C polymorphism is associated with increased blood TG and lower HDL-C, and is not associated with increased LDL-C [50]. The different effects of the -1131 T>C polymorphism on TC, TG, HDL-C, and LDL-C suggest that pathogenesis may different in different lipid fractions and the -1131 T>C polymorphism may exert varying effect.

Significant heterogeneity was respectively found across the studies for TG, LDL-C, and HDL-C. Prominent sources of heterogeneity include: differences in ancestry, different study design, gender difference, and healthy status of the subjects included in the meta-analysis, etc. To explore the potential source of the observed heterogeneity, we performed subgroup analyses stratified by the characteristics of the subjects. For TG, the heterogeneity was effectively decreased or removed in the subgroup analyses. For LDL-C and HDL-C, significant heterogeneity was still observed in some subgroups. The sources of heterogeneity were further evaluated by Galbraith plot. Outlier studies were identified as the main contributors of heterogeneity by using the plot. The heterogeneity was effectively removed or decreased after exclusion of these outlier studies.

Significant publication bias was present in the studies on TG. Any meta-analysis carries the risk of publication bias caused by the fact that small studies with positive results are more likely to be published than those with negative results [51]. Therefore, a meta-analysis of larger studies will generally yield more conservative results than a meta-analysis of all published studies [51]. To further evaluate the relationship between the APOA5 -1131 T>C polymorphism and blood TG, we carried subgroup analysis only including the studies with sample size more than five hundreds. There was no evidence of publication bias for this subgroup analysis. The results from this analysis also suggest a significant association of the -1131 T>C polymorphism with lower levels of TG.

The association of the APOA5 -1131 T>C polymorphism with blood TC, TG, and HDL-C is not likely to be due to type I errors (false-positive results). First, the random effects model was used in our meta-analysis. Because the random effects model provides a more conservative evaluation of the significance of the association than one based on fixed effects, the results from our meta-analysis are based on a more conservative evaluation, which was expected to avoid false-positive results. Second, 37 studies with 37859 subjects were included in this meta-analysis. Among the subjects, 36% of them were carriers of -1131C allele. If the incidence of the Thr 54 carriers was sufficiently high, this may have prevented the type I error.

The possible mechanism by which -1131 T>C polymorphism modulates lipids metabolism has not yet been investigated in detail. Study has shown that APOA5 knockout mice develop hypertriglyceridemia, whereas transgenic mice overexpressing APOA5 have low triglyceride levels [5]. Since the -1131 T>C polymorphism is located within the promoter region of the APOA5 gene and might potentially result in a decreased rate of APOA5 mRNA translation, which could lead to lower plasma APOA5 levels, and then cause to higher levels of TG [32]. It is well known that plasma levels of TG and HDL-C are inversely related. A higher blood TG commonly associates with a lower blood HDL-C. A hypothesis for this association is that the delayed TG hydrolysis, due APOA5 deficiency, reduces the availability of surface components of TG-rich lipoproteins (which contribute to HDL-C formation), thereby leading to a decreased formation of HDL-C [52]. Another hypothesis for the association of the APOA5 -1131 T>C polymorphism with lower HDL-C is that the absence of APOA5, which is a minor protein component of HDL-C, renders HDL-C more unstable and more easily removed from the circulation [52]. Our meta-analysis also suggests the significant association of the -1131 T>C polymorphism with lower TC. It is more likely that the -1131 T>C polymorphism is in linkage disequilibrium with other causative mutations or nearby genes involved in the metabolism of TC [19]. APOA5 gene was identified as part of the *APOA1/C3/A4/A5 *gene cluster which is key components in modulating lipid metabolism [5,11]. Several SNPs in the APOA1/C3/A4/A5 gene cluster have been reported to significantly affect blood lipid levels [23,24]. Studies have reported linkage disequilibrium of the -1131 T>C polymorphism with other variants located in the *APOA1/C3/A4/A5 *gene cluster [19].

The current meta-analysis has several limitations which should be noted. First, hyperlipidemia originates from the interactions of multiple genes, environmental factors, and behavior. Lacking of the original data of the included studies limited our further evaluation of potential interactions because the interactions among gene-gene, gene-environment and even different polymorphic loci of the same gene may modulate blood lipid levels. For example, we did not perform the stratification analyses by environmental factors such as diet, exercise, and smoking. A more precise analysis could be conducted if more detailed individual data were available, which would allow for an adjusted estimate. Second, we did not perform a meta-analysis for haplotype analysis of the APOA5 gene, because a very limited number of studies were available. Third, for LDL-C, a relative small number of subjects were included in this meta-analysis, which may lead to lower statistic power and type II errors (false- negative results). More studies with larger population will be required to further examine the association. Fourth, because it was very difficult to get the full papers published in various languages, we only included the studies published in English. Thus the limitations mentioned may affect our final conclusions.

## Conclusions

In conclusion, our meta-analysis supports the strong association of the APOA5 -1131 T>C polymorphism with higher levels of TC and TG, and lower levels of HDL-C.

## Competing interests

The authors declare that they have no competing interests.

## Authors' contributions

TFZ participated in the design, data acquisition and analysis, interpretation of results, and manuscript writing. JPZ participated in the data acquisition and analysis. Both authors read and approved the final manuscript.

## Pre-publication history

The pre-publication history for this paper can be accessed here:

http://www.biomedcentral.com/1471-2350/11/120/prepub

## Supplementary Material

Additional file 1 **A figure describing the flow of candidate and eligible papers**. Click here for file

Additional file 2 **Figure legends of additional file **1**, additional file **4**, and additional file **5. Click here for file

Additional file 3 **Supplementary tables**. Two supplementary tables including: Table S1. Characteristics of individual studies included in the meta-analysis. Table S2. Blood lipid levels by genotypes of individual studies included in the meta-analysis. Click here for file

Additional file 4 **Galbraith plot detecting potential sources of heterogeneity**. Click here for file

Additional file 5 **Funnel plot detecting potential publication bias**. Click here for file
