# Supplementary figures and images for: Association of the apolipoprotein A5 gene -1131 T>C polymorphism with fasting blood lipids: a meta-analysis in 37859 subjects
Source: BMC Med Genet. 2010 Aug 10;11:120. doi: 10.1186/1471-2350-11-120 (PMC2924867; doi:10.1186/1471-2350-11-120)

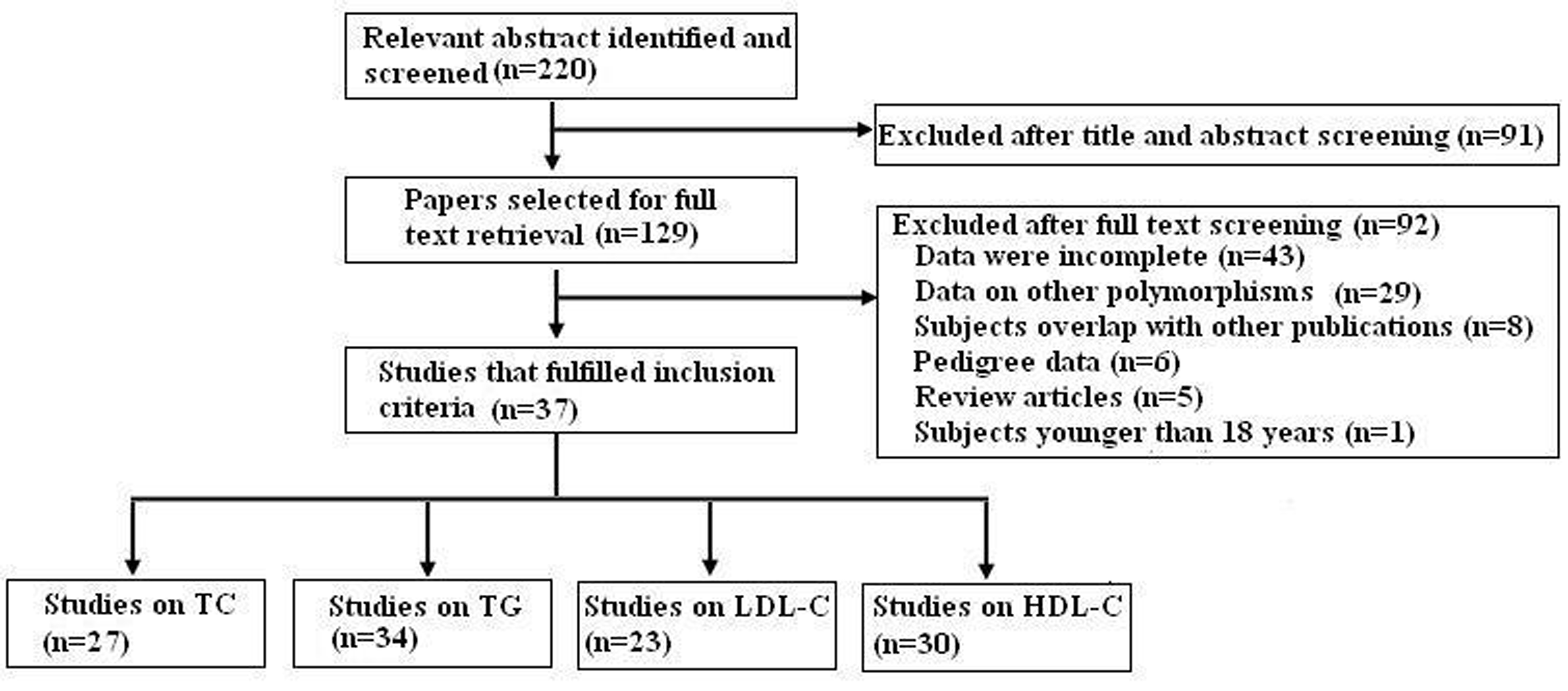

Supplement: Additional file 1 — A figure describing the flow of candidate and eligible papers. [file 1471-2350-11-120-S1.JPEG]

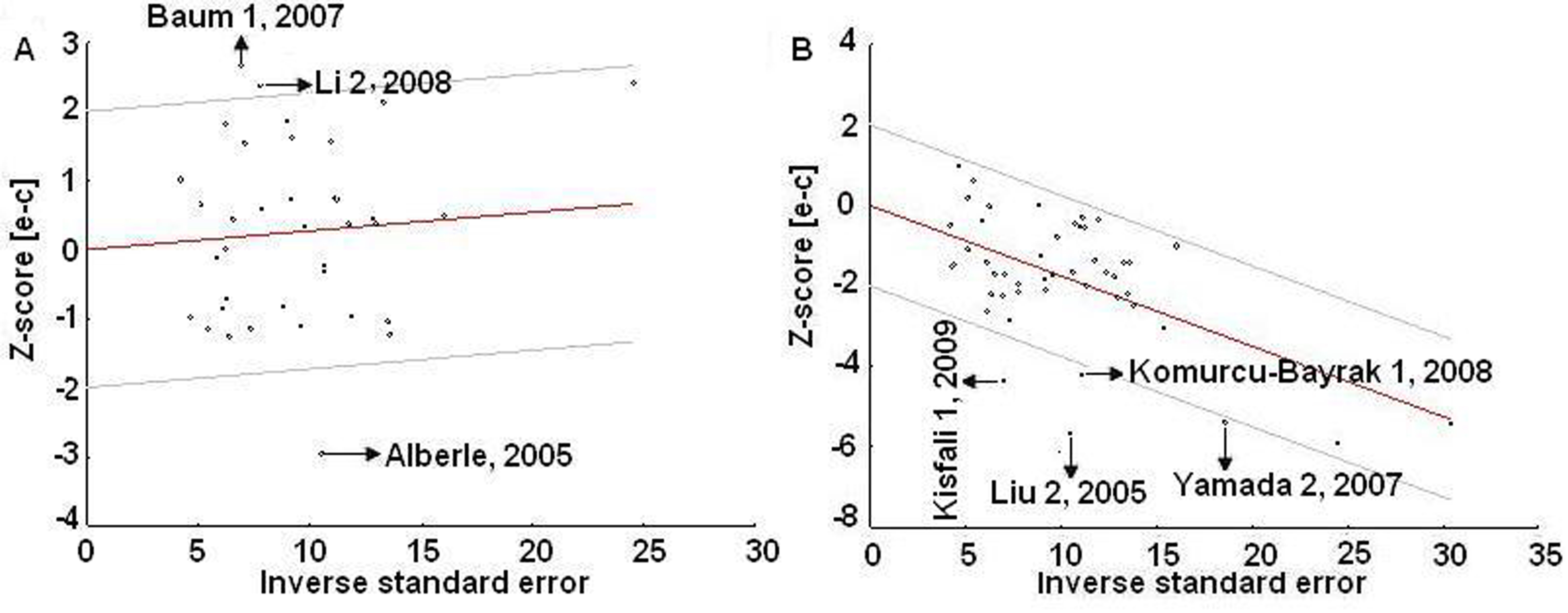

Supplement: Additional file 4 — Galbraith plot detecting potential sources of heterogeneity. [file 1471-2350-11-120-S4.JPEG]

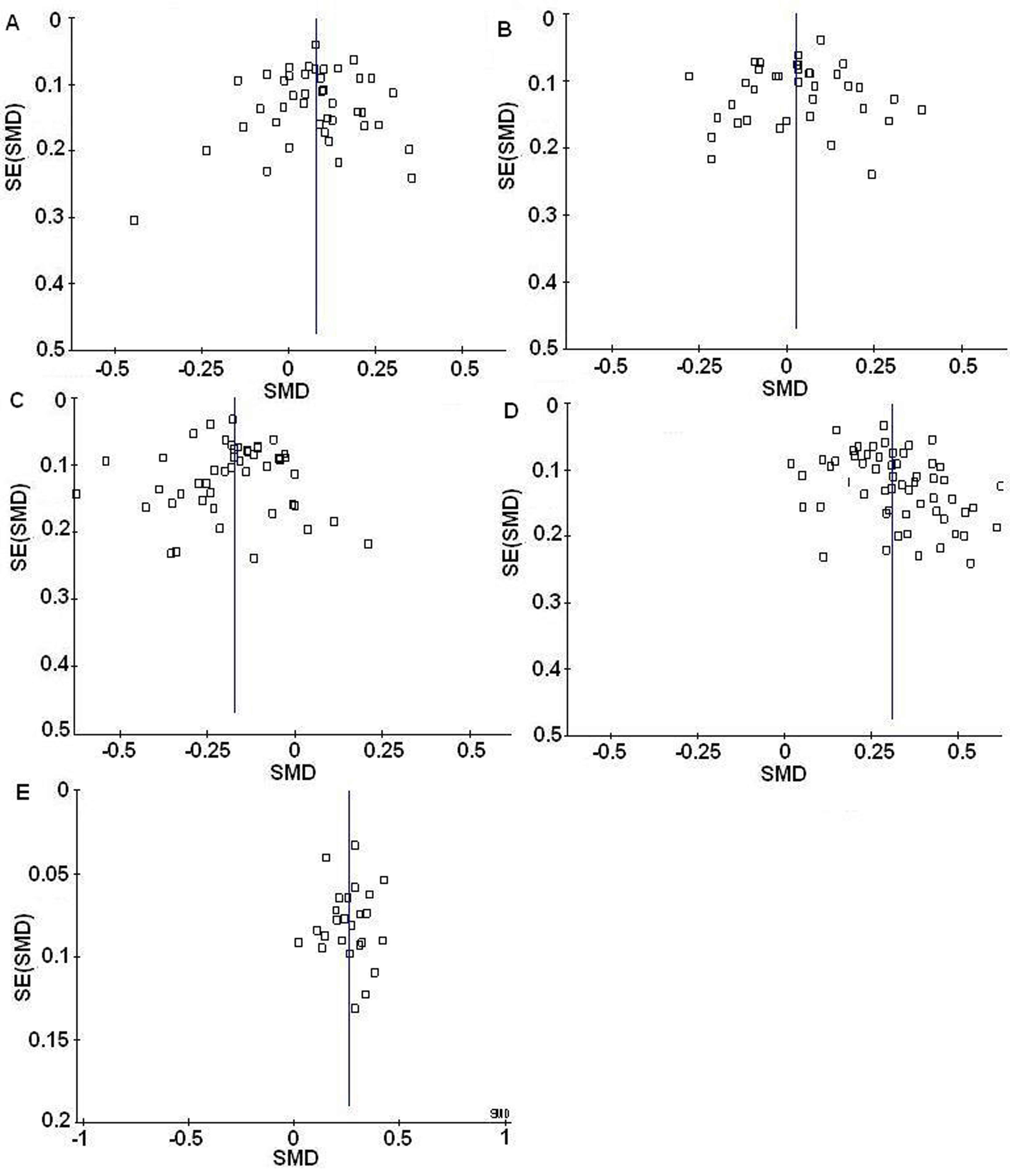

Supplement: Additional file 5 — Funnel plot detecting potential publication bias. [file 1471-2350-11-120-S5.JPEG]
