# Supplementary material for: Association of the apolipoprotein A5 gene -1131 T>C polymorphism with fasting blood lipids: a meta-analysis in 37859 subjects
Source: BMC Med Genet. 2010 Aug 10;11:120. doi: 10.1186/1471-2350-11-120 (PMC2924867; doi:10.1186/1471-2350-11-120)
Supplement: Additional file 2 — Figure legends of additional file 1, additional file 4, and additional file 5. [file 1471-2350-11-120-S2.DOC]

**Additional figure legends**

**Additional file 1 Flowchart outlining the process of search criteria and study selection.**

**Additional file 4 Galbraith plot analysis to evaluate heterogeneity.** (A) LDL-C; (B) HDL-C. For each point, the Z-score is plotted against the inverse of the standard error. The least precise results from small studies appear towards the left of the plot and the results from the largest studies appear toward the right. The dotted lines positioned two units above and below the solid line delimit the area, in which, in the absence of heterogeneity, 95% of the points would be expected to lie. Reference numbers identify the studies that lie outside the 95% confidence limits.

**Additional file 5 Funnel plots for publication bias test.** Each point represents a separate study for the indicated association. SE(SMD), standard error of the standardized mean difference. (A) The funnel plot comparing the differences in TC; (B) The funnel plot comparing the differences in LDL-C; (C) The funnel plot comparing the differences in HDL-C; (D) The funnel plot comparing the differences in TG (including all studies on TG); (E) The funnel plot of larger studies on TG.
