# Supplementary material for: Association of the apolipoprotein A5 gene -1131 T>C polymorphism with fasting blood lipids: a meta-analysis in 37859 subjects
Source: BMC Med Genet. 2010 Aug 10;11:120. doi: 10.1186/1471-2350-11-120 (PMC2924867; doi:10.1186/1471-2350-11-120)
Supplement: Additional file 3 — Supplementary tables. Two supplementary tables including: Table S1. Characteristics of individual studies included in the meta-analysis. Table S2. Blood lipid levels by genotypes of individual studies included in the meta-analysis. [file 1471-2350-11-120-S3.DOC]

**Table S1: Characteristics of individual studies included in the meta-analysis**

| First author, year, reference | Ethnicity | Gender | Take lipid-lowering medication | Study population | Outcomes |
| --- | --- | --- | --- | --- | --- |
| Járomi 1, 2009 [11]  Járomi 2, 2009 [11]  Maasz 1, 2008 [13]  Maasz 2, 2008 [13]  Kisfali 1, 2009 [14] ^a^  Kisfali 2, 2009 [14]  Hahne, 2008 [9]  Charriere, 2008 [15]  Sundl, 2007 [12]  Chandak 1, 2006 [16]  Martinelli, 2007 [17]  Elosua, 2006 [6]  Hodoglugil 1, 2006 [18]  Hodoglugil 2, 2006 [18]  Alberle, 2005 [35] ^b^  Szalai 1, 2004 [19] ^c^  Szalai 2, 2004 [19]  Talmud, 2004 [20]  Pennacchio 1,2002 [36]  Pennacchio 2,2002 [36]  Girona, 2008 [21]  Hubacek 1, 2008 [40] ^c^  Hubacek 2, 2008 [40]  Huang, 2008 [22]  Jang 1, 2009 [10]  Jang 2, 2009 [10]  Chien, 2008 [23]  Yamada 1, 2007 [24]  Yamada 2, 2007 [24] ^a^  Yamada 3, 2007 [24]  Yamada 4, 2007 [24]  Hsu, 2006 [26]  Liu 1, 2005 [7]  Liu 2, 2005 [7] ^a^  Bi 1, 2004 [27]  Bi 2, 2004 [27]  Jang, 2004 [28]  Li, 2004 [29]  Baum, 2003 [41]  Li 1, 2008 [30]  Li 2, 2008 [30] ^b^  Li 3, 2008 [30]  Hsu, 2008 [31]  Yan 1, 2005 [32]  Yan 2, 2005 [32]  Yan 3, 2005 [32]  Baum 1, 2007 [42] ^b^  Baum 2, 2007 [42]  Baum 3, 2007 [42]  Lai 1, 2003 [33] ^c^  Olano-Martin, 2008 [37]  Chandak 2, 2006 [16]  Hodoglugil 3, 2006 [18]  Hodoglugil 4, 2006 [18]  Aouizerat, 2003 [34]  Pennacchio 3,2002 [36]  Pennacchio 4,2002 [36]  Pennacchio 5,2002 [36]  Pennacchio 6,2002 [36]  Komurcu-Bayrak 1, 2008 [8] ^a^  Komurcu-Bayrak 2, 2008 [8]  Chaaba 1, 2005 [38]  Chaaba 2, 2005 [38]  Mattei, 2009 [39]  Lai 2, 2003 [33]  Lai 2, 2003 [33] | European  European  European  European  European  European  European  European  European  European  European  European  European  European  European  European  European  European  European  European  European  European  European  East Asian  East Asian  East Asian  East Asian  East Asian  East Asian  East Asian  East Asian  East Asian  East Asian  East Asian  East Asian  East Asian  East Asian  East Asian  East Asian  East Asian  East Asian  East Asian  East Asian  East Asian  East Asian  East Asian  East Asian  East Asian  East Asian  East Asian  Other  Other  Other  Other  Other  Other  Other  Other  Other  Other  Other  Other  Other  Other  Other  Other | M/F  M/F  M/F  M/F  M/F  M/F  M/F  M/F  M  M/F  M/F  M/F  M  F  M  M/F  M/F  M  F  M  M/F  M  F  F  M/F  M/F  M/F  M/F  M/F  M/F  M/F  M/F  M/F  M/F  M/F  M/F  M  M/F  M  M/F  M/F  M/F  M/F  M/F  M/F  M/F  M/F  M/F  M/F  M/F  M/F  M/F  M  F  M/F  F  M  F  M  F  M  M/F  M/F  M/F  M/F  M/F | NA  NA  NA  NA  NA  NA  Partly used  Partly used  NA  NA  No  NA  No  No  NA  No  No  NA  NA  NA  NA  NA  NA  NA  Partly used  No  No  NA  NA  NA  No  NA  No  No  No  No  No  No  NA  NA  NA  NA  NA  No  No  No  No  Partly used  Partly used  NA  No  NA  No  No  Ceased lipid- lowering medication for at least 1 month  NA  NA  NA  NA  No  No  No  No  Partly used  NA  NA | Ischemic stroke patients  Control subjects with negative brain MRI findings  Patients with ischemic stroke  Individuals free from neuro-imaging alterations and clinical history of stroke  Metabolic syndrome patients  Healthy subjects  Obese patients and non-obese subjects  Type 2 diabetic patients  Healthy nonsmoking subjects  Parents from the Plymouth EarlyBird study  Subjects with CAD and subjects without CAD  Framingham Offspring Study  Random subjects  Random subjects  Hyperlipaemic and overweight men  Patients with severe CAD  Subjects without CAD  Subjects with CHD  Random sample of Dallas County residents  Random sample of Dallas County residents  Non-smoker type 2 diabetic patients  MONICA study  MONICA study  Hypertriglyceridemic and control subjects  Coronary artery disease patients  Healthy control subjects  Individuals free of hypertriglyceridemia  Random subjects visiting hospital  Community-dwelling elderly individuals  Subjects with metabolic syndrome  Control subjects excluding related diseases  Subjects free of CAD  Control subjects  Patients with CHD  Patients with CHD  Apparently healthy individuals  Healthy men  Volunteers without hyperlipoproteinemia, hypertension, diabetes, endocrine or metabolic disorders  Subjects with high or low triglyceride  Healthy subjects  Type 2 diabetes  Type 2 diabetic patients with cerebral infarction  Subjects with metabolic syndrome and subjects without metabolic syndrome  Healthy controls  Type 2 diabetes  Type 2 diabetic with CHD  Normal controls  Type 2 diabetic patients without diabetic nephropathy  Type 2 diabetes patients with diabetic nephropathy  Random subjects  Healthy adults  Parents from the Pune children study  Random subjects  Random subjects  Subjects with combined hyperlipidemia, subjects with hypoalphalipoproteinemia, subjects with hyper alphalipoproteinemia, and control subjects  Random sample of Dallas County residents  Random sample of Dallas County residents  Random sample of Dallas County residents  Random sample of Dallas County residents  Random subjects  Random subjects  Type 2 diabetes  Subjects without type 2 diabetes  Random older adults  Random subjects  Random subjects | TG  TG  TC, TG  TC, TG  TC, TG, HDL-C  TC, TG, HDL-C  TG, HDL-C  TC, TG, LDL-C, HDL-C  TC, TG, LDL-C, HDL-C  TC, TG, LDL-C, HDL-C  TC, TG, LDL-C, HDL-C  TC, TG, LDL-C, HDL-C  TG  TG  TC, TG, LDL-C, HDL-C  TC, TG, LDL-C, HDL-C  TC, TG, LDL-C, HDL-C  TC, TG  TG  TG  TC, TG, LDL-C, HDL-C  TC, LDL-C, HDL-C  TC, LDL-C, HDL-C  TC, TG  TC, TG, LDL-C, HDL-C  TC, TG, LDL-C, HDL-C  TG  TG, HDL-C  TG, HDL-C  TG, HDL-C  TG, HDL-C  TC, TG, LDL-C, HDL-C  TC, TG, LDL-C, HDL-C  TC, TG, LDL-C, HDL-C  TC, TG, LDL-C, HDL-C  TC, TG, LDL-C, HDL-C  TC, TG, LDL-C, HDL-C  TC, TG, LDL-C, HDL-C  TC, LDL-C, HDL-C  TC, TG, LDL-C, HDL-C  TC, TG, LDL-C, HDL-C  TC, TG, LDL-C, HDL-C  TG, HDL-C  TC, TG, LDL-C, HDL-C  TC, TG, LDL-C, HDL-C  TC, TG, LDL-C, HDL-C  LDL-C, HDL-C  LDL-C, HDL-C  LDL-C, HDL-C  TC, TG, LDL-C, HDL-C  TC, TG, LDL-C, HDL-C  TC, TG, LDL-C, HDL-C  TG  TG  TC, TG, LDL-C, HDL-C  TG  TG  TG  TG  TG, HDL-C  TG, HDL-C  TC, TG, HDL-C  TC, TG, HDL-C  TC, TG, LDL-C, HDL-C  TC, TG, LDL-C, HDL-C  TC, TG, LDL-C, HDL-C |

NA: not available, CAD: coronary artery disease, MONICA study: Multinational Monitoring of Trends and Determinants in Cardiovascular Diseases study, M: male, F: female.

^a^ Outlier studies for HDL-C.

^b^ Outlier studies for LDL-C.

^c^ The study was found to deviate from HWE.

**Table S2: Blood lipid levels by genotypes of individual studies included in the meta-analysis**

| First author, year | Genotype | | | TC ^e^ | | TG ^e^ | | LDL-C ^e^ | | HDL-C ^e^ | |
| --- | --- | --- | --- | --- | --- | --- | --- | --- | --- | --- | --- |
|  | TC/CC | TT | Total | TC/CC | TT | TC/CC | TT | TC/CC | TT | TC/CC | TT |
| Járomi 1, 2009 [11]  Járomi 2, 2009 [11]  Maasz 1, 2008 [13]  Maasz 2, 2008 [13]  Kisfali 1, 2009 [14]  Kisfali 2, 2009 [14]  Hahne, 2008 [9]  Charriere, 2008 [15]  Sundl, 2007 [12]  Chandak 1, 2006 [16]  Martinelli, 2007 [17]  Elosua, 2006 [6]  Hodoglugil 1, 2006 [18]  Hodoglugil 2, 2006 [18]  Alberle, 2005 [35]  Szalai 1, 2004 [19]  Szalai 2, 2004 [19]  Talmud, 2004 [20]  Pennacchio 1,2002 [36]  Pennacchio 2,2002 [36]  Girona, 2008 [21]  Hubacek 1, 2008 [40]  Hubacek 2, 2008 [40]  Huang, 2008 [22]  Jang 1, 2009 [10]  Jang 2, 2009 [10]  Chien, 2008 [23]  Yamada 1, 2007 [24]  Yamada 1, 2007 [24]  Yamada 2, 2007 [24]  Yamada 3, 2007 [24]  Yamada 4, 2007 [24]  Hsu, 2006 [26]  Liu 1, 2005 [7]  Liu 2, 2005 [7]  Bi 1, 2004 [27]  Bi 2, 2004 [27]  Jang, 2004 [28]  Li, 2004 [29]  Baum, 2003 [41]  Li 1, 2008 [30]  Li 2, 2008 [30]  Li 3, 2008 [30]  Hsu, 2008 [31]  Yan 1, 2005 [32]  Yan 2, 2005 [32]  Yan 3, 2005 [32]  Baum 1, 2007 [42]  Baum 2, 2007 [42]  Baum 3, 2007 [42]  Lai 1, 2003 [33]  Olano-Martin, 2008 [37]  Chandak 2, 2006 [16]  Hodoglugil 3, 2006 [18]  Hodoglugil 4, 2006 [18]  Aouizerat, 2003 [34]^a^  Pennacchio 3,2002 [36]  Pennacchio 4,2002 [36]  Pennacchio 5,2002 [36]  Pennacchio 6,2002 [36]  Komurcu-Bayrak 1, 2008 [8]^b^  Komurcu-Bayrak 2, 2008 [8]^c^  Chaaba 1, 2005 [38]  Chaaba 2, 2005 [38]  Mattei, 2009 [39]  Lai 2, 2003 [33]  Lai 3, 2003 [33] | 79  13  69  12  61  28  25  64  46  19  100  296  23  66  148  42  33  54  48  42  25  162  212  202  359  421  119  2146  2141  802  636  396  359  256  302  175  181  73  199  96  168  156  144  317  72  96  72  89  196  180  1181  45  188  381  319  185  154  111  62  36  164  147  36  25  184  326  210 | 434  159  309  119  282  256  84  336  251  218  516  1971  208  505  458  174  277  242  311  286  144  957  1086  114  382  320  163  1640  1652  617  381  375  318  246  181  94  136  85  135  71  172  100  76  298  83  76  41  109  186  187  1235  214  336  1220  983  442  537  376  125  103  528  532  100  76  618  334  298 | 513  172  378  131  343  284  109  400  297  237  616  2267  231  571  606  216  310  296  359  328  169  1119  1298  316  741  741  282  3786  3793  1419  1017  771  677  502  483  269  317  158  334  167  340  256  220  615  155  172  113  198  382  367  2416  259  524  1601  1302  627  691  487  187  139  692  679  136  101  802  660  508 | -  -  5.77±1.25  4.79±0.69  5.51±1.11  5.18±1.06  -  5.33±0.89  5.06±0.878  202.6±23.5  226±44  212±41  -  -  264.9±63.5  6.18±0.75  5.59±0.50  5.26±0.63  -  -  4.79±0.63  5.77±1.00  5.94±1.18  187.66±41.10  198±34.36  172±40.77  -  -  -  -  -  -  206±43  5.22±0.82  5.51±0.80  5.35±0.86  5.03±1.06  191.7±37.04  178.30±43.74  5.19±0.78  4.79±0.93  4.96±0.94  5.25±1.12  -  4.93±0.95  5.11±0.90  4.93±1.15  -  -  -  5.49±1.20  5.64±0.84  164.0±35.2  -  -  264±84  -  -  -  -  -  -  5.17±1.07  4.53±0.99  4.70±1.13  5.69±1.55  5.66±1.20 | -  -  5.79±1.23  5.22±0.98  5.30±1.06  5.42±1.00  -  5.42±1.11  4.82±0.932  189.8±36.9  222±41  205±37  -  -  275.2±71.3  6.10±0.79  5.52±0.62  5.18±0.73  -  -  4.68±0.79  5.72±1.04  5.78±1.14  187.25±36.65  196±36.55  172±40.96  -  -  -  -  -  -  202±36  5.03±0.78  5.52±0.76  5.31±1.10  4.98±0.98  188.5±34.76  165.90±37.96  5.22±0.93  4.69±1.08  4.84±0.98  5.02±1.04  -  4.73±0.88  5.00±0.87  4.56±0.88  -  -  -  5.39±1.41  5.77±1.02  156.6±36.7  -  -  264±77  -  -  -  -  -  -  5.17±1.15  4.61±1.32  4.77±1.09  5.57±1.64  5.54±1.38 | 1.92±0.80  1.84±0.43  2.14±1.08  2.00±1.04  2.90±1.55  1.66±0.54  1.87±1.51  2.24±1.06  1.21±0.582  125.3±54.9  186±104  172±135  166±80  142±79  377.6±156.6  2.22±0.62  1.82±0.79  1.85±1.00  135±97  213±175  2.14±1.29  -  -  132.58±92.00  155±91  164±106.91  102.3±39.8  1.78±1.66  -  1.75±1.04  2.25±1.54  1.18±0.76  161±155  1.74±0.87  2.13±0.89  2.41±2.07  1.85±1.31  148.41±95.6  165.89±147.0  -  1.61±0.25  1.78±0.38  1.94±0.35  1.79±1.45  1.29±0.84  1.83±1.40  1.73±1.33  -  -  -  1.58±1.48  1.82±1.01  109.7±55.9  174±120  122±88  230±267  100.32±76  150±158  167±101  252±186  1.83±0.86  2.14±1.04  2.62±1.59  1.75±1.07  1.95±1.82  1.95±1.87  1.93±1.08 | 1.70±0.62  1.51±0.50  1.68±0.53  1.48±0.55  2.33±1.28  1.38±0.34  1.51±0.67  1.98±1.15  1.05±0.522  95.3±56.1  154±80  137±91  139±93  120±76  356.7±161.2  1.95±0.58  1.48±0.52  1.56±0.67  125±98  164±134  1.69±0.94  -  -  101.39±63.94  129±58.63  135±71.55  80.9±29.7  1.39±0.84  -  1.36±0.73  1.96±1.03  1.05±0.54  129±107  1.39±0.78  1.73±0.89  1.84±1.34  1.32±0.89  113.7±60.9  112.72±79.1  -  1.52±0.32  1.65±0.33  1.76±0.41  1.43±1.17  0.92±0.57  1.18±0.87  1.16±0.43  -  -  -  1.34±1.76  1.59±0.73  91.5±56.8  144±99  104±64  198±198  99±75  140±206  158±201  173±150  1.56±0.88  1.86±1.07  2.05±1.61  1.6±1.42  1.80±1.23  1.56±2.01  1.67±1.21 | -  -  -  -  -  -  -  3.39±0.85  2.89±0.652  123.9±24.0  151±40  129±35  -  -  165.7±53.9  3.89±0.95  3.47±0.65  -  -  -  2.68±0.76  3.68±0.98  3.78±1.12  -  117±32.38  96.3±39.51  -  -  -  -  -  -  124±37  3.07±0.96  3.20±0.88  3.37±0.96  3.00±0.83  112.0±28.6  94.29±28.2  3.19±0.78  2.59±0.42  2.81±0.44  3.05±0.52  -  3.11±0.85  3.22±0.79  3.15±0.99  3.24±0.85  3.26±0.93  3.59±1.18  3.48±1.18  3.58±0.83  101.5±32.5  -  -  173±68  -  -  -  -  -  -  -  -  2.73±0.95  3.77±1.39  3.83±1.19 | -  -  -  -  -  -  -  3.53±0.90  2.68±0.728  115.5±35.4  148±37  128±32  -  -  181.8±58.5  3.91±0.95  3.59±0.55  -  -  -  2.83±0.69  3.65±0.95  3.61±1.04  -  120±33.62  99.3±36.31  -  -  -  -  -  -  123±31  3.01±0.89  3.22±0.83  3.30±0.90  3.07±0.60  115.3±29.2  88.73±24.4  3.35±0.84  2.51±0.49  2.67±0.48  2.94±0.46  -  3.11±0.82  3.17±0.69  3.03±0.82  2.92±0.81  3.23±0.82  3.73±1.22  3.37±1.05  3.72±1.02  96.8±33.0  -  -  175±62  -  -  -  -  -  -  -  -  2.80±0.84  3.72±1.46  3.75±1.21 | -  -  -  -  1.22±0.04  -  1.39±0.38  0.97±0.24  1.33±0.296  54.2±10.0  48±15  50±16  -  -  42.7±12.9  1.29±0.14  1.28±0.16  -  -  -  1.15±0.31  1.21±0.37  1.47±0.37  -  49.2±12.50  43.4±11.25  -  -  1.32±0.47  1.45±0.37  1.12±0.32  1.61±0.32  51±15  1.65±0.85  1.03±0.45  1.12±0.33  1.34±0.36  50.0±17.0  53.20±14.69  1.15±0.39  1.54±0.31  1.25±0.33  1.06±0.27  1.40±0.39  1.33±0.36  1.35±0.36  1.07±0.31  1.51±0.44  1.37±0.39  1.14±0.31  1.34±0.31  1.24±0.38  40.8±11.8  -  -  55±19  -  -  -  -  1.17±0.26  1.02±0.24  0.83±0.34  0.83±0.26  1.15±0.33  1.21±0.46  1.13±0.39 | -  -  -  -  1.24±0.03  -  1.52±0.38  1.08±0.29  1.33±0.308  55.8±14.0  51±15  51±16  -  -  44.6±11.7  1.30±0.16  1.26±0.18  -  -  -  1.09±0.28  1.25±0.34  1.51±0.37  -  50.5±12.31  45.2±10.91  -  -  1.40±0.42  1.56±0.39  1.19±0.40  1.67±0.35  54±19  1.69±0.81  1.28±0.48  1.20±0.29  1.34±0.36  50.1±10.1  55.31±15.72  1.28±0.34  1.62±0.38  1.34±0.32  1.13±0.32  1.45±0.35  1.48±0.34  1.44±0.31  1.06±0.22  1.64±0.36  1.40±0.36  1.20±0.35  1.42±0.35  1.34±0.44  41.4±12.8  -  -  59±24  -  -  -  -  1.26±0.23  1.03±0.23  0.90±0.32  0.92±0.25  1.16±0.33  1.28±0.55  1.14±0.35 |
| Total ^d^ | 13633 24226 37859 | | | | | | | | | | |

Data were expressed as mean±SD.

^a^156 carriers of -1131C allele and 408 subjects with genotype TT were included for assaying LDL-C levels, and 181 carriers of -1131C allele and 436 subjects with genotype TT were included for assaying HDL-C levels.

^b^152 carriers of -1131C allele and 488 subjects with genotype TT were included for assaying TG levels.

^c^134 carriers of -1131C allele and 462 subjects with genotype TT were included for assaying TG levels.

^d^ Among the 37859 subjects, 64% had the genotype TT, and 36% were carriers of -1131C allele.

^e^ Different units were used in the studies included in this meta-analysis.

^f^ 34193, 20961, 19860, and 29588 subjects were included in comparing the difference in blood TG, TC, LDL-C, and HDL-C, respectively.
